# The complete chloroplast genome of *Lindera pulcherrima* var. *hemsleyana* (Lauraceae)

**DOI:** 10.1080/23802359.2020.1810164

**Published:** 2020-08-31

**Authors:** Shao-Juan Qian, Yong-Hong Zhang

**Affiliations:** School of Life Sciences, Yunnan Normal University, Kunming, China

**Keywords:** *Lindera pulcherrima* var. *hemsleyana*, chloroplast genome, phylogenetic analysis, Lauraceae

## Abstract

*Lindera pulcherrima* var. *hemsleyana* is an evergreen tree of the genus *Lindera* with important medicinal value. The complete chloroplast genome of *L. pulcherrima* var. *hemsleyana* is 152,710 bp in length, containing a LSC region of 93,751 bp, a SSC region of 18,813 bp, and a pair of inverted repeats (IRA and IRB) of 20,073 bp each. The genome encoded 128 genes, including 83 protein-coding genes, 8 ribosomal RNA genes, 36 transfer RNA genes, and one pseudogene (*ycf1*). Most of these genes occurred in a single copy, whereas 15 genes occurred in double copies, including all rRNA, 6 tRNA, and 5 protein-coding genes. The GC content of the whole genome, LSC, SSC, and IR regions is 39.2%, 38.0%, 33.9%, and 44.4%, respectively. A total of 90 SSRs were discovered, the numbers of mono-, di-, tri-, tetra-, penta-, and hexa-nucleotides SSRs were 66, 10, 4, 8, 1, and 1, respectively. Phylogenetic analysis of cp genomes from 24 species of Lauraceae revealed that cp genomes of *L. pulcherrima* var. *hemsleyana*, *L. pulcherrima* var. *attenuata*, and *L. pulcherrima* formed a monophyletic clade with 100% bootstrap value.

*Lindera pulcherrima* (Nees) J. D. Hooker (Lauraceae), a dominant evergreen tree in shady forests, is an important medicinal plant distributed in southern China, Nepal, and India. The active compounds contained in *L. pulcherrima* have good anti-inflammatory, antiviral, antibacterial, and antitumor pharmacological activities (Joshi and Mathela 2012). *L. pulcherrima* var. *hemsleyana* is distinguished with other two varieties, *L. pulcherrima* var. *attenuate* and *L. pulcherrima* var. *pulcherrima*, for its elliptic leaf blade, glabrous ovary, and glabrous young fruits (Tsui et al. 1982). The leaves and bark are used as a spice in cold, fever, and cough (Joshi and Mathela 2012). Its quality wood is widely used in furniture and architecture. Though phylogenic studies on Lauraceae were conducted, some taxonomic controversies on intra-generic relationship of the genus *Lindera* as well as the inter-generic relationship with allied genera were still unsolved (Tian et al. 2019).

The whole chloroplast genome was extracted from the fresh leaves of a healthy *L. pulcherrima* var. *hemsleyana* tree with modified CTAB protocol (Yang et al. 2014), it was collected from the natural habitats of Yongshan County (28°05′13.20″N, 103°35′38.40″E), Yunnan province, China. The voucher specimen (ELK-254) was deposited in the Herbarium of Yunnan Normal University (YNUB). Paired-end reads were sequenced by using Illumina HiSeq 2500-PE150 (Illumina, San Diego, CA). All raw reads were filtered through NGS QC toolkit_v2.3.3 to get clean reads with default parameters (Patel and Jain 2012). The NOVOPlasty was used to de novo assemble the clean reads to form the chloroplast genome (Dierckxsens et al. 2017). The assembled structures and genes of the complete cp genome were annotated with Geneious 20.0.3 (Kearse et al. 2012). The online tRNAscan-SE service was used to verify tRNA genes of the *L. pulcherrima* var. *hemsleyana* (Schattner et al. 2005).

The chloroplast genome of *L. pulcherrima* var. *hemsleyana* (GenBank accession no.: MT801093) was a quadripartite circular with 152,710 bp in size with total GC content 39.2%. A pair of inverted repeat (IR) region (20,073 bp) separated the large single copy (LSC) region (93,751 bp) and small single copy (SSC) region (18,813 bp). The GC content of the LSC, SSC, and IR regions is 38.0%, 33.9%, and 44.4%, respectively. There are 128 genes predicted, including 81 protein-coding genes, 36 tRNA genes, eight rRNA genes, and one pseudogene (*ycf1*). IMEx (Mudunuri and Nagarajaram 2007) was used to identify the SSRs with minimum repeat number set to 10, 5, 4, 3, 3, and 3. A total of 90 SSRs were discovered, the numbers of mono-, di-, tri-, tetra-, penta-, and hexa-nucleotides nucleotides SSRs were 66, 10, 4, 8, 1, and 1, respectively.

In order to understand the phylogenetic relationship between *L. pulcherrima* var. *hemsleyana* and related taxa of the genus *Lindera*, 19 taxa of *Lindera* and five species of Subfamily Lauroideae that are downloaded from NCBI as outgroups were used to construct a molecular phylogenetic tree. Twenty-four cp genomes were aligned by MAFFT 7.308 (Katoh and Standley 2013). The maximum likelihood (ML) tree was constructed with RAxML 8.2.11 (Stamatakis 2014) under the nucleotide substitution model of GTR + G. The bootstrap values were calculated from 1000 replicates analysis. The phylogenetic topology (Figure 1) of *L. pulcherrima* is consistent with the morphological delimitation of this species. *L. pulcherrima* var. *hemsleyana* and other two varieties formed one monophyletic clade with 100% bootstrap value. The complete chloroplast genome of *L. pulcherrima* var. *hemsleyana* will provide useful genetic resource for further study on phylogeny of the genus *Lindera* and Lauraceae.

**Figure 1.**
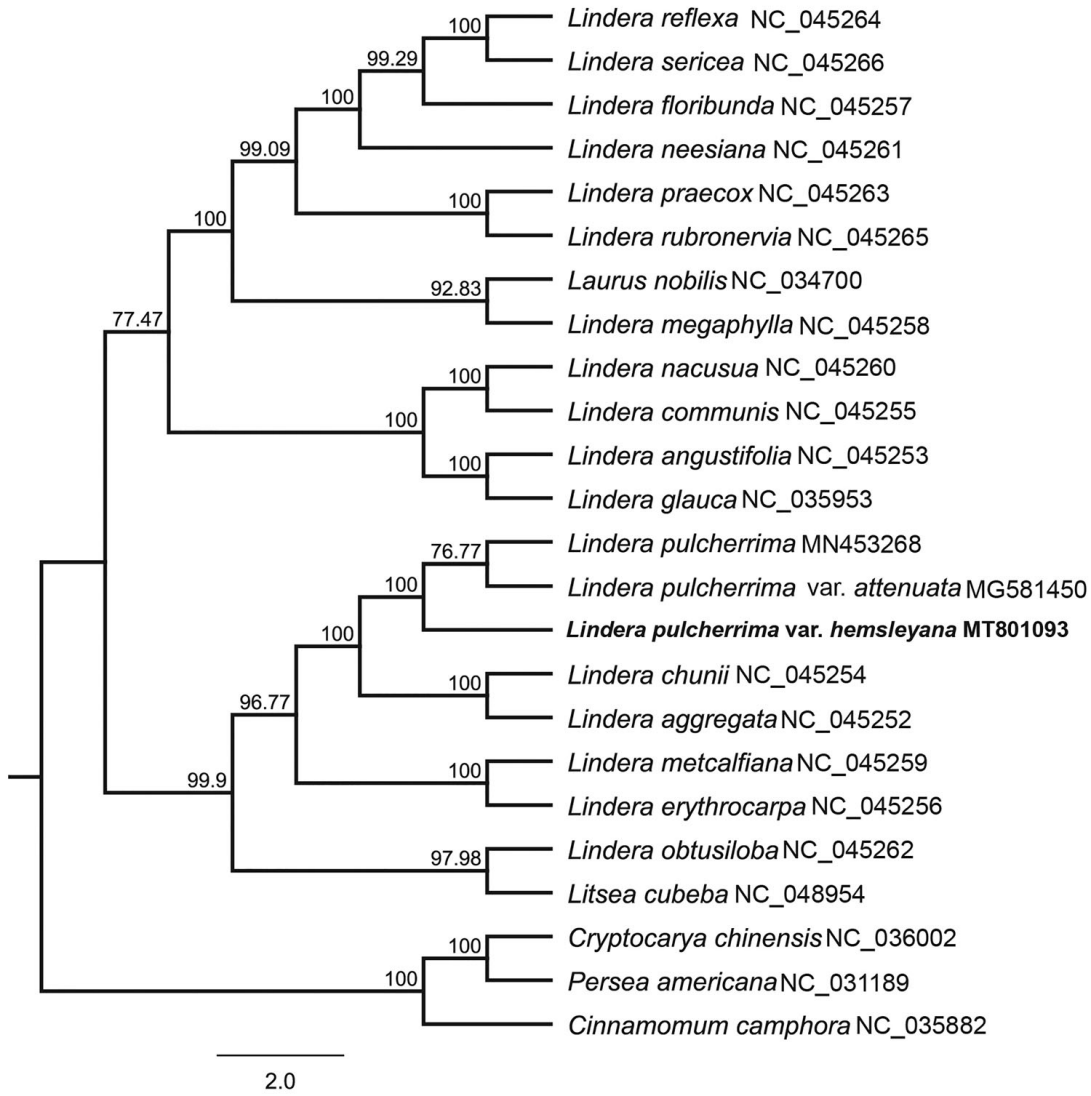
Molecular phylogenetic tree of 24 species of Lauraceae based on complete plastome sequences with maximum likelihood analysis.

## Data Availability

The data that support the findings of this study are openly available in NCBI at https://www.ncbi.nlm.nih.gov/, reference number [MT801093], or available from the corresponding author.
